# Acupuncture at homotopic acupoints exerts dual effects on bladder motility in anesthetized rats

**DOI:** 10.1186/s12906-015-0781-6

**Published:** 2015-08-08

**Authors:** Qingguang Qin, Qian Mo, Kun Liu, Xun He, Xinyan Gao, Bing Zhu

**Affiliations:** Institute of Acupuncture and Moxibustion, China Academy of Chinese Medical Sciences, Beijing, 100700 China; Department of Acupuncture and Moxibustion, Henan Orthopaedics Hospital, Luoyang, 471002 Henan Province China; Department of Acupuncture, Guang’anmen Hospital, China Academy of Chinese Medical Sciences, Beijing, 100053 China

**Keywords:** Manual acupuncture (MA), Bladder activity, Intravesical pressure, Acupoint, Dual effects, Spinal segmentation

## Abstract

**Background:**

In Chinese medicine, dual effects on target organs are considered a primary characteristic of acupoint. Acupoints may be classified as heterotopic or homotopic in terms of spinal segmental innervation: homotopic acupoints contain afferent innervation in the same segment from which efferent fibers innervate target visceral organs, and heterotopic acupoints utilize different spinal segments to innervate target visceral organs than the segment receiving the afferent signal. It is well-known that dual effects of acupuncture on the bladder can be generated based on different states of the bladder, however, the dual effects of single acupoint stimulation and acupoint site-specificity (homotopic acupoints and heterotopic acupoints) on the bladder have yet to be investigated.

**Methods:**

Twenty Sprague-Dawley rats were anesthetized and the intravesical pressure was measured via a manometric balloon inserted into the bladder. The acupuncture needle was separately inserted to a depth of 4 mm at the acupoints RN1 (Huiyin), RN3 (Zhongji), BL28 (Pangguangshu), BL32 (Ciliao), RN2 (Qugu) or BL23 (Shenshu), and manually rotated right then left with a frequency of 2 Hz for 1 min. Following acupuncture stimulation, bladder pressure was recorded and compared against the pre-stimulation measurements.

**Results:**

During the bladder’s active state, manual acupuncture (MA) at RN1, RN3, BL28, BL32 or RN2 inhibited bladder motility (*P* < 0.01). In the static bladder, MA at RN1, RN3, BL28, BL32, RN2 or BL23 increased bladder motility (*P* < 0.01).

**Conclusions:**

MA at homotopic acupoints may produce dual effects on bladder motility: inhibiting bladder motility when in an active state and enhancing bladder motility when in a static state.

## Background

Acupuncture, a nonspecific physical stimulation, restores normal functions by facilitating or inhibiting the inherent regulatory system in the body, and not by directly acting on the pathogen. In Chinese medicine, acupuncture is generally believed to balance the energy flows of Yin and Yang, the two major “negative” and “positive” forces governing the body [1–3]. It is also believed that acupuncture exerts differential effects on internal organs to restore the homeostatic balance. For example, previous studies suggest the site-specific inhibitory or stimulatory effects of acupuncture on gastric motility [4, 5].

The location of acupoints plays an essential role in acupuncture’s effects on visceral organ functions. Generally, acupoints may be classified as heterotopic and homotopic points, in terms of spinal segmental innervation patterns. Our previous studies have demonstrated that acupuncture at homotopic acupoints, where afferent innervation is in the same spinal segment from which the efferents innervate visceral organs, decreases intragastric pressure. Conversely, acupuncture at heterotopic acupoints, which utilize different spinal segments to innervate a visceral organ has been shown to induce gastrointestinal facilitation in complete spinal rats [6].

Parasympathetic fibers are the primary form of innervation to and from the urinary bladder. Presynaptic fibers arise from neurons in spinal cord segments S2–4 and project via pelvic splanchnic nerves and the inferior hypogastric and vesical plexuses to the bladder. They form synapses with postsynaptic neurons that are found on or near the bladder wall. Parasympathetic fibers provide motor innervation to the detrusor muscle and inhibit the internal urethral sphincter. Sympathetic fibers arise from neurons in spinal cord segments T11–L3, project via lumbar splanchnic nerves, and synapse on hypogastric plexuses [7]. Sympathetic nerves play little role in bladder motor activity, but they do appear to heavily innervate the neck and trigone of the bladder. Sympathetic stimulation allows for bladder neck closure, which is crucial for bladder filling. Somatic fibers to the external urethral sphincter arise from motor neurons in spinal cord segments S2–4 and project to the bladder via the pudendal nerve [8].

A key characteristic of acupoint stimulation is that of “dual effects,” defined as the capability of acupuncture at the same acupoint to effectively inhibit overexcited functions or amplify deficient functions in the same target organ [9, 10]. Although the dual effects of acupuncture on the gastrointestinal tract or heart are controversial, it is well-known that dual effects of acupuncture on the urinary bladder may be generated based on differential activity states of the bladder. Acupuncture-like stimulation to the perineal area may increase activity (emptying) of the over-filling bladder, and decrease overactive emptying of the half-filling bladder [6, 11]. These findings led us to propose the following questions: Can stimulating a single homotopic or heterotopic acupoint regulate the bladder in a dual fashion? Do acupoints exhibit dual effects on the urinary bladder in a site-specific manner?

Here, we selected the homotopic acupoints RN1 (Huiyin), RN3 (Zhongji), BL28 (Pangguangshu), BL32 (Ciliao), and RN2 (Qugu), and the heterotopic acupoint BL23 (Shenshu), because these acupoints are most frequently utilized to treat human bladder disorders in acupuncture clinics. We wished to verify the hypothesis that depending on the bladder’s state, stimulating a single homotopic or heterotopic acupoint will produce dual effects on bladder activity in anesthetized rats.

## Methods

### Animal preparation

Animal experiments were carried out in accordance with the National Institutes of Health’s Guide for the Care and Use of Laboratory Animals and approved by the Institutional Animal Care and Use Committee of China Academy of Chinese Medical Sciences. Twenty adult male Sprague-Dawley rats (250-300 g) were purchased from the Laboratory Animal Center of the Academy of Military Medical Sciences. For one week prior to the experiment, all animals were housed in groups of 3-4 rats with *ad* libitum access to food and water and a 12 h: 12 h light-dark cycle (dark cycle 8:00 PM-8:00 AM). All rats were anesthetized with an intraperitoneal injection of urethane (1.0-1.2 g/kg, Sigma-Aldrich, St Louis, USA) and supplementary anesthesia was administered if limb withdrawal or a fluctuation of heart rate was observed. Core body temperature was monitored and maintained at 37.0 ± 0.5 °C by a feedback-controlled electric blanket (ALC-HTP, Shanghai Alcott Biotech CO., Ltd, China).

### Intravesical pressure recording

As previously reported [12], bladder motility was assessed via cystometrogram, which measures pressure within the bladder while being slowly filled (0.05 ml/min) with warm water using a transurethral catheter. Briefly, an incision was made medially down the abdomen and the bladder was exposed. A manometric balloon was inserted into the bladder, filled with approximately 0.5 mL warm water, and was connected to polyethylene tubing, providing a pressure of approximately 100 mmH_2_O. Intravesical bladder pressure was measured with a transducer through the polyethylene tube and was recorded using a Mac Lab system (AD Instruments, Australia). At around 100 mmH_2_O, the bladder in an anesthetized rat switches automatically between active (lasting 10-13 min) and static state (lasting 3-5 min). Thirty minutes after recording a stable intravesical pressure (at either the active or static state), we performed acupuncture stimulation that lasted 1 min long, on different acupoints in random order.

### Acupuncture stimulation

In this study, we selected the homotopic acupoints RN1, RN3, BL28, BL32, and RN2, and the heterotopic acupoint BL23. These acupoints are most frequently targeted to treat human bladder disorders in acupuncture clinics. According to “*Chinese Acupuncture and Moxibustion*” [13], RN1 is between the anus and the root of the scrotum in males and between the anus and the posterior labial commissure in females; RN3 is on the anterior midline, 10 mm below the umbilicus; BL28 is 4-5 mm lateral to midline, at the level of the second posterior sacral foramen; BL32 is on the sacrum, medial and inferior to the posteriosuperior iliac spine, just at the second posterior sacral foramen; RN2 is on the midpoint of the upper border of the symphysis pubis; and BL23 is 4-5 mm lateral to midline, at the level of the lower border of the spinous process of the second lumbar vertebra. Acupuncture needles (0.25 mm in diameter and 13 mm in length, Suzhou Hwato Medical Instruments, China) were manually inserted unilaterally, vertically, or slightly obliquely (as necessary) to a depth of approximately 4 mm and rotated right, then left, at a frequency of 2 Hz for 1 min. In the present study, manual acupuncture (MA) was performed at the above acupoints in a random order. After baseline intravesical pressure was recovered following completion of one acupoint stimulation, the next acupoint was stimulated.

### Statistical analysis

Data analysis was based on previously reported techniques [14]. Quantification of bladder motility was determined by calculating the motility index (MI). The MI is equivalent to the area under the curve of the motility recording, where seconds are represented on the x-axis and intravesical pressure (mmH_2_O) is represented on the y-axis. MI was calculated using a computer-assisted system (Power Lab, AD Instruments). The mean MI for 30 min prior to MA was expressed as 100 %, and MA-induced changes in bladder pressure were normalized to this baseline for each acupoint stimulated. Statistical analysis was performed with SPSS19.0 software (IBM, USA). All data are displayed as mean ± SEM. For significance evaluation, data sets with normal distributions were analyzed by a paired *t*-test. *P*-values <0.05 was considered statistically significant.

## Results

### Acupuncture at homotopic acupoints decreases the intravesical pressure in the active state

Firstly, we observed that a baseline internal pressure of bladder was 20-50 mmH_2_O, as recorded with the 0.5 mL inflating water balloon. Bladder motility included the active state and the static state. In the active state, the amplitude of the contractile wave induced by the balloon was 10-30 mmH_2_O, wave duration was 10-30 s, motility index (MI) was 573.86 ± 199.45 mmH_2_O*s, and frequency was 10.10 ± 4.27 units/min. In the static state, the amplitude of the contractile wave was less than 2 mmH_2_O, wave duration was less than 10 s, MI was less than 25.30 ± 17.62 mmH_2_O*s, and frequency was 8.50 ± 2.21 units/min.

**Fig. 1:**
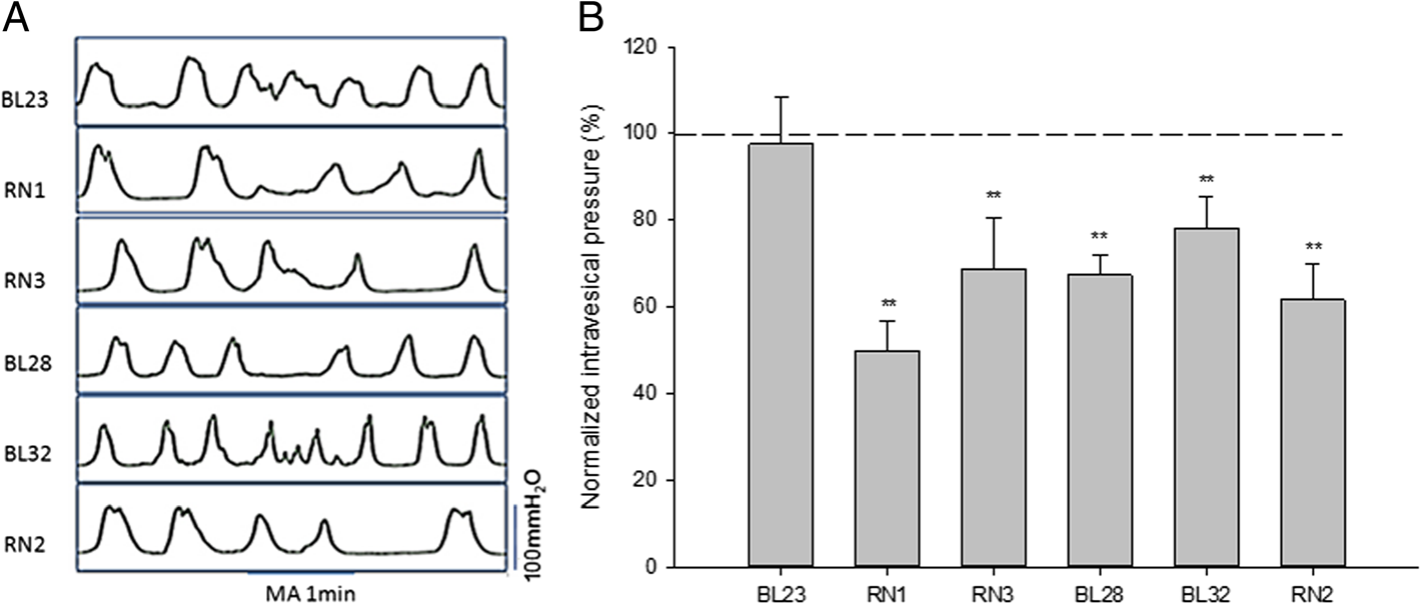
Effects of MA on intravesical pressure in the active state (**a**) Representative traces of bladder motility with or without acupuncture in the active state of bladder. **b** The intravesical pressure was significantly decreased by MA at RN1, RN3, BL28, BL32, RN2 individually (**P < 0.01, paired *t* test, *n* = 20), but was not significantly altered by acupuncture at BL23 in active state of bladder. The intravesical pressure after acupuncture was normalized to the pre-acupuncture baseline, shown as a dashed line and set to 100 %

The robust contractile wave stabilized in the bladder’s active period before acupuncture. As shown in Fig. 1, compared to the pre-acupuncture intravesical pressure, manual acupuncture (MA) at the homotopic acupoints RN1, RN3, BL28, BL32 or RN2 significantly decreased the intravesical pressure (49.80 ± 6.96 % of pre-MA baseline, 68.63 ± 12.08 % of pre-MA baseline, 67.27 ± 4.82 % of pre-MA baseline, 78.17 ± 7.21 % of pre-MA baseline and 61.53 ± 8.22 % of pre-MA baseline, respectively. *P* < 0.01). Conversely, the intravesical pressure was not significantly affected after MA at the heterotopic acupoint BL23; (97.63 ± 10.55 % of pre-MA baseline, *P* > 0.05).

### Acupuncture at either homotopic or heterotopic acupoints increased the intravesical pressure in the static state

**Fig. 2:**
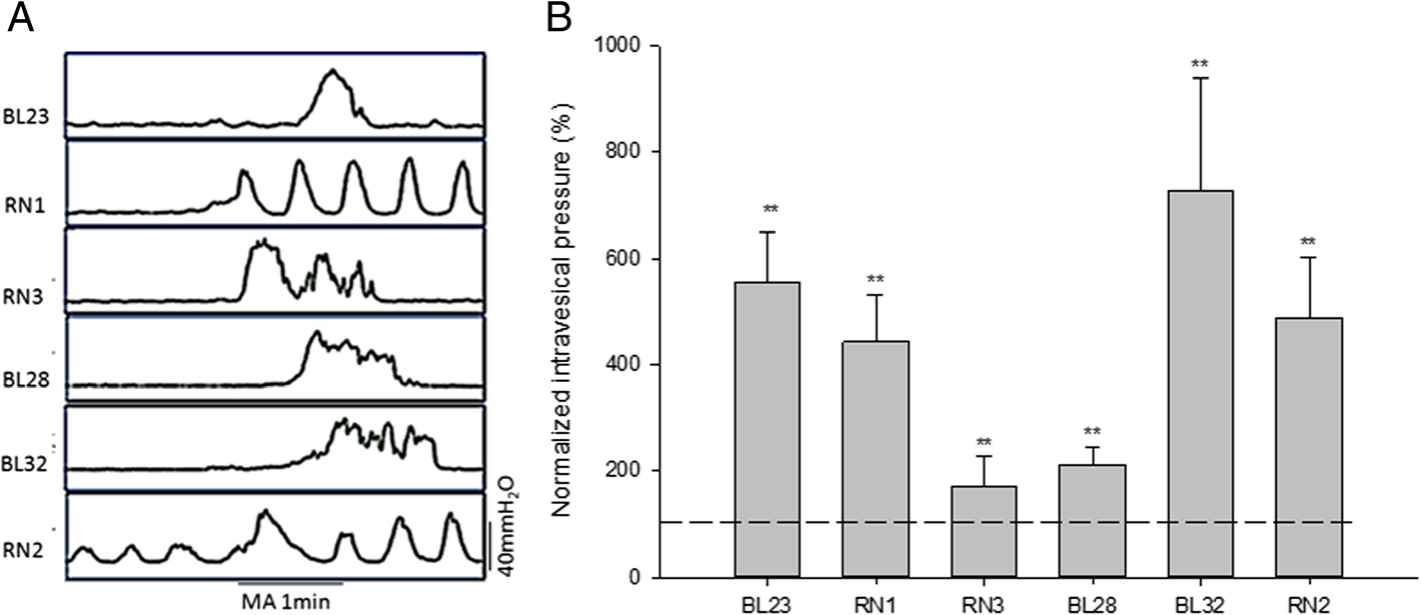
Effects of MA on intravesical pressure in the static state (**a**) Representative traces of bladder motility with or without acupuncture in the static state of bladder. **b** The intravesical pressure was significantly increased by MA at RN1, RN3, BL28, BL32, RN2 and BL23 individually in the static state of bladder (**P < 0.01, paired *t*-test, *n* = 20). The intravesical pressure after acupuncture was normalized to the pre-acupuncture baseline, shown as a dashed line and set to 100 %

As shown in Fig. 2, when the bladder was in the static state, MA at each acupoint in this experiment significantly increased the intravesical pressure compared to the pre-acupuncture baseline. Post-acupuncture MIs were as follows: RN1 443.85 ± 87.63 %, RN3 171.49 ± 55.76 %, BL28 211.07 ± 32.66 %, BL32 727.52 ± 211.43 %, RN2 488.43 ± 114.19 %, and BL23 554.64 ± 93.65 % of pre-MA baseline (*P* < 0.01).

### MA at homotopic acupoints produced dual effects on the intravesical pressure

**Fig. 3:**
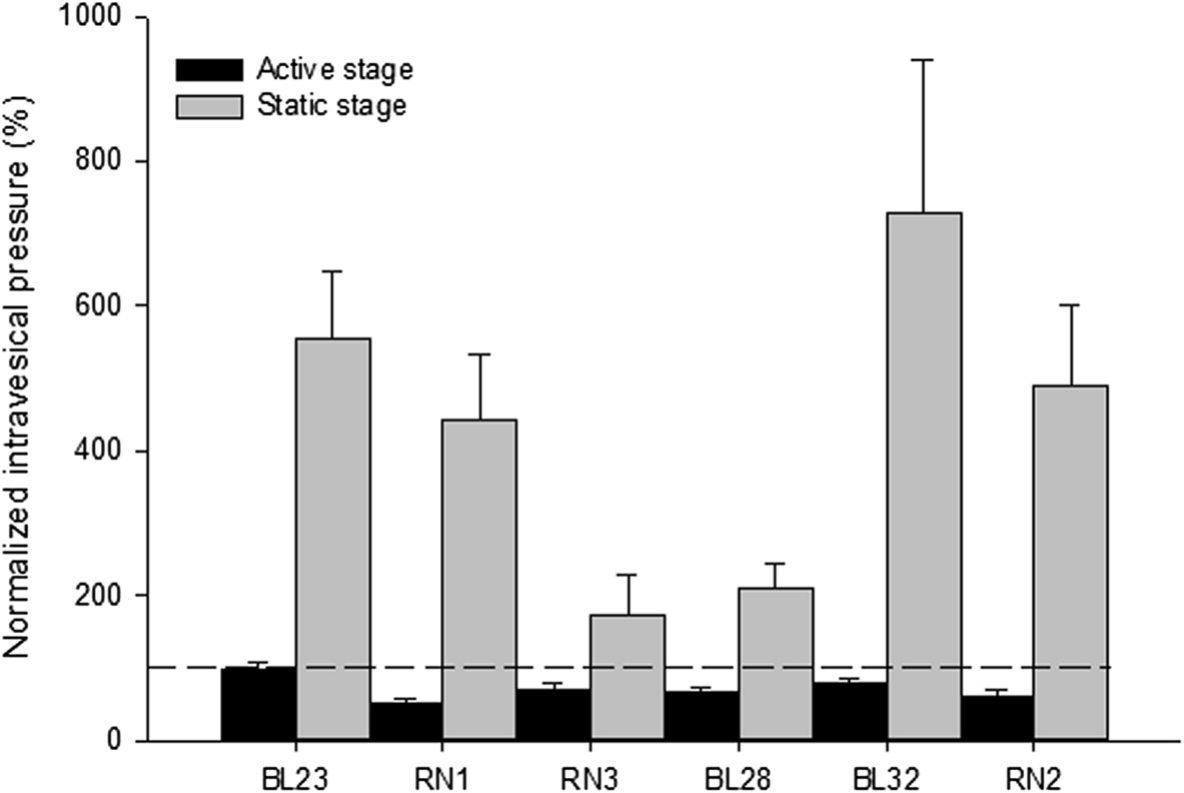
Dual effects of MA on intravesical pressure MA at RN1, RN3, BL28, BL32 and RN2 produced significant dual effects on intravesical pressure, decreasing the pressure in the active state and increasing the pressure in the static state. MA at BL23 increased intravesical pressure only in the static state. The intravesical pressure after acupuncture was normalized to the pre-acupuncture baseline, shown as a dashed line and set to 100 %

Finally, we compared the effects of MA at each acupoint on bladder motility in different states. As shown in Fig. 3, MA at the homotopic acupoints RN1, RN3, BL28, BL32 and RN2 significantly decreased the intravesical pressure in the active state, but significantly increased intravesical pressure in the static state of bladder. Post-acupuncture MIs were as follows: RN1: 49.80 ± 6.96 % of pre-baseline for active state *vs* 443. 85 ± 87.63 % of pre-baseline for static state; RN3: 68.63 ± 12.08 % of pre-baseline for active state *vs* 171.49 ± 55.76 % of pre-baseline for static state; BL28: 67.27 ± 4.82 % of pre-baseline for active state *vs* 211.07 ± 32.66 % of pre-baseline for static state; BL32: 78.17 ± 7.21 % of pre-baseline for active state *vs* 727.52 ± 211.43 % of pre-baseline for static state; RN2: 61.53 ± 8.22 % of pre-baseline for active state *vs* 488.43 ± 114.19 % of pre-baseline for static state. MA at BL23 only induced a significant increase of intravesical pressure in the static state of bladder (MI = 97.63 ± 10.55 % of pre-baseline for active state *vs* 554.64 ± 93.65 % of pre-baseline for static state). These data suggest that MA at homotopic acupoints, which are afferently innervated by the same or adjacent spinal cord segment which efferently innervates the bladder, can produce dual effects on urinary bladder motility depending on the initial condition of the bladder.

## Discussion

In the present study, after the bladder was filled with 0.5 mL water, it was half-full, which approximates to the residual urine volume. We observed the effect of acupuncture on bladder motility during the active period and static state of the detrusor and found that acupuncture at the homotopic acupoints RN1, RN3, BL28, BL32 and RN2 significantly decreased the bladder motility during the active stage of the detrusor muscle, whereas acupuncture at the heterotpic acupoint BL23 did not significantly alter bladder motility. We also found that acupuncture at the above acupoints increased bladder motility during the static stage of the detrusor muscle. These results suggest that acupuncture at these homotopic acupoints could produce dual effects on bladder motility which are dependent on the initial bladder conditions.

Somato-visceral convergence in the dorsal and ventral horn of the spinal cord has been recognized for many years [15–22]. It has been well documented that the somatosensory inputs from the skin and/or muscle of a particular spinal segment are involved in the control of various autonomic functions [23, 24]. For example, stimulating the skin at certain points excites the activity of pelvic nerve discharge and increases bladder motility when it is empty, or inhibits pelvic nerve discharge activity to decrease bladder motility when it is filled. However, the transection of pelvic nerves diminished these reflexes [25]. Parasympathetic nerves of the pelvis play an important role in the somato-visceral reflex, whereas sympathetic nerves play little to no role in this reflex [26, 27]. The motor neurons of the primary micturition center are located in the anterior horn of the sacral spinal cord segments S2, S3 and S4. The motor nerves (parasympathetic pelvic splanchnic nerves) derived from this primary center terminate in ganglia and internal sphincter of the bladder, and control contraction of the detrusor muscle and relaxation of internal anal sphincter to enhance bladder emptying. The afferent nerve fibers from the bladder muscle project into the spinal cord primarily through parasympathetic fibers (pelvic nerves). The signal from the mucous layer of the bladder is mainly transduced through sympathetic fibers [28, 29] which exit from the lateral horn of spinal cord segments T10, L1, and L2 to relax the detrusor. On the other hand, exciting parasympathetic fibers originating from the anterior horn of spinal cord segments S2, S3, and S4 causes the detrusor to contract [30].

Nociceptive stimulation of skin, particularly when it involves afferents which share spinal segments in common with the pelvic outflow, can elicit excitatory and inhibitory effects on the bladder depending on the degree of bladder distension [9, 25–27, 31–33]. In the current study, the sensory nerve of the homotopic acupoints RN1, RN3, and RN2 in the male rat’s perineal area originate from the branches of the pudendal nerve (scrotal nerve) [34]. The afferent fibers of the pudendal nerve are somatic afferent nerves, and the afferent fibers of pelvic nerve are visceral afferent fibers; these two fiber types overlap in spinal segments L5-S1 [35]. The afferent fibers from the acupoint BL23 project to spinal segments L1-3; the afferent fibers from BL28 project to the neurons of segments L2-S5 [36]; the neurons which innervate BL32 are located at spinal segments L2-S4, most densely in S2-S4 [37]; and the afferent fibers from BL28 project to segments L4-S3 [38]. The fibers that innervate RN1, RN3, BL28, and RN2 overlap with fibers in the lumbosacral motor center of bladder. Our findings suggest that acupuncture at the above acupoints may produce dual effects on the bladder activities under different functional states, verifying that only stimulating the same or adjacent segment of spinal cord which innervates the bladder could produce dual effects on bladder activities. On the contrary, the nerve fiber innervation of the heterotopic acupoint BL23 is not in the same segments as the primary parasympathetic micturition center (S2-S4), and acupuncture at this acupoint thus only caused an excitatory effect on the bladder in the static state. Similar findings were observed in MA of other heterotopic points LI11 (Quchi), SP6 (Sanyinjiao) and GB34 (Yanglingquan) (data not shown). Our findings provide valuable information for MA treatment of functional bladder disorders. We note that the results of our study differ from a similar prior study by Morrison et al. [39] on heterotopic stimulation. In our study, stimulation of the heteropic point BL-23 produced no effect on bladder contractility when the bladder was active but lead to excitatory effect when the bladder was static. In contrast, Morrison et al. found stimulating a heterotopic point around the tibial nerve produced no effect when the bladder pressure is low (static state), and lead to both excitatory and inhibitory effects when the bladder pressure is high (active state). The difference between our study and that by Morrison et al. could be explained by differences in the degree of artificial bladder extension, the location of the point stimulated, and the mode of stimulation.

## Conclusions

Under different physiological conditions, manual acupuncture at the homotopic acupoints produced dual effects on the urinary bladder, inhibiting bladder motility when in an active state and enhancing bladder motility when in a static state.
